# A81 OPTIMIZING THE INDICATIONS FOR BILIARY STENT PLACEMENT IN PATIENTS WITH CBD STONES: A QUALITY IMPROVEMENT INITIATIVE TO ENHANCE PATIENT CARE AND REDUCE HEALTHCARE RESOURCE UTILIZATION

**DOI:** 10.1093/jcag/gwac036.081

**Published:** 2023-03-07

**Authors:** I Alzahrani, S I S Alhaidari, M Alhanaee, A Decanini, M Mohamed, S Zepeda-Gomez, P Mathura, J Zhang, G Sandha

**Affiliations:** 1 Medicine, University of Alberta, Edmonton, Canada; 2 Medicine, Imam Abdulrahman bin Faisal University, Dammam, Saudi Arabia; 3 Alberta Health Services, Edmonton, Canada

## Abstract

**Background:**

A retrospective chart audit was performed to review biliary stent utilization from January 2020 to 2021 at the University of Alberta Hospital (UAH). Inappropriate stent usage was identified in 16% of patients with common bile duct (CBD) stones presenting for endoscopic retrograde cholangiopancreatography (ERCP). To improve this clinical practice, a quality improvement (QI) initiative was developed and completed.

**Purpose:**

To reduce the number of inappropriately inserted biliary stents in patients with CBD stones.

**Method:**

The results of the chart audit (pre-intervention) were shared with the ERCP group. The QI intervention was to align biliary stent insertion in accordance with published guidelines. A chart audit (post-intervention) was then performed on all ERCPs from July, 2021 to June, 2022. The indication for biliary stent insertion was assessed independently by two blinded reviewers.

**Result(s):**

A total of 661 patients (337 F) with mean age of 59±19 years (range 12-98 years) underwent 885 ERCPs during this post-intervention period. Of the 661 patients, 384 (58%) were referred for CBD stones. A total of 192 biliary stents (105 plastic, 85 metal) were placed during the first ERCP (192/661, 29%), as compared to the pre-intervention year (223/598, 37%, *p=ns*). However, only 13/192 stents (7%) were placed not in accordance with published guidelines (*kappa*=0.53), compared with 63/223 (28%) in the pre-intervention year (*p<0.0001*). This accounts for a 75% reduction in overall unnecessary stent placement. This reduction was mainly seen in the CBD stone subgroup, where there was an 88% reduction in inappropriate biliary stent placement compared to the pre-intervention year (8/384, 2% vs. 61/376, 16%, *p<0.0001*).

**Image:**

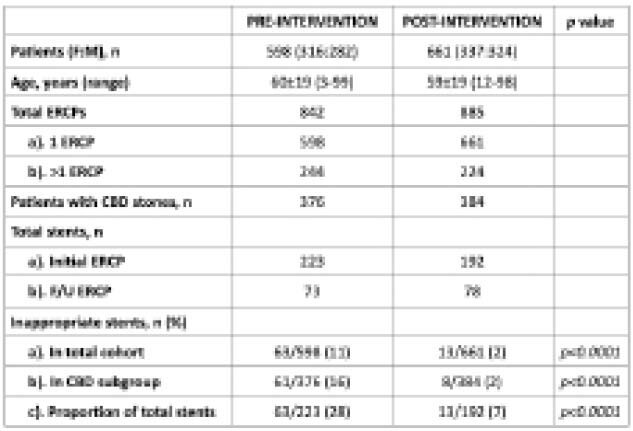

**Conclusion(s):**

Education to align practice in accordance with published guidelines has demonstrated a significant improvement in biliary stent insertion during ERCP in patients with CBD stones. This has resulted in significantly fewer inappropriate stent placements, a reduction in unnecessary follow-up ERCPs, and an overall saving of healthcare resources.

**Please acknowledge all funding agencies by checking the applicable boxes below:**

None

**Disclosure of Interest:**

None Declared

